# More than just a bed: mental health service users’ experiences of self-referral admission

**DOI:** 10.1186/s13033-016-0045-y

**Published:** 2016-02-25

**Authors:** Turid Møller Olsø, Camilla Buch Gudde, Inger Elise Opheim Moljord, Gretha Helen Evensen, Dag Øivind Antonsen, Lasse Eriksen

**Affiliations:** Norwegian Resource Centre for Community Mental Health, NTNU Social Research AS, Trondheim, Norway; Forensic Department Brøset, Centre for Research and Education in Forensic Psychiatry, St. Olavs University Hospital, P.O. 1803, Lade, NO-7440, Trondheim, Norway; Nidaros Community Mental Health Centre, St. Olav’s University Hospital, Trondheim, Norway; Department of Neuroscience, Faculty of Medicine, Norwegian University of Science and Technology, Trondheim, Norway; Resource Centre for Service User Experience and Service Development (KBT Middle-Norway), Mental Health, NO-7409, Trondheim, Norway

**Keywords:** User involvement, Mental health services, Self-referral, Mental illness, User experiences

## Abstract

**Background:**

Several community mental health centres and mental hospitals in Norway now allow users with a diagnosis of severe mental illness to self-refer for admission. This give a group of service users who are well-known to service providers the opportunity to refer themselves for short inpatient stays without contacting their doctor, a duty doctor or emergency department. Evidence on self-referral admissions is lacking.

**Aim:**

To explore service users’ experiences of having the opportunity to refer themselves for a short inpatient stay.

**Methods:**

Forty-two qualitative semi-structured interviews were undertaken between 2010 and 2014 in a group of 28 service users with serious mental illness and with or without substance abuse problems. All respondents had a contract which allowed them to self-refer for inpatient treatment. Systematic text condensation was applied in the analyses.

**Results:**

Self-referral inpatient admission is more than just a bed. It was perceived as a new, unconventional health service, which differed substantially from earlier experiences of inpatient care and was characterised by different values and treatment principles. The differences were related to the content, quality and organisation of treatment. Having the option to decide about admission for oneself and having access to services focusing on individual needs seem to enhance service users’ confidence, both in the services they use and in their own ability to cope with everyday life.

**Conclusions:**

Self-referral inpatient admission is a concrete example of how a user involvement policy can be implemented in mental health services. It is important to emphasise that the self-referral admission process described here is an offer in development and that we are awaiting findings from a larger RCT study. More evidence is needed to determine what aspects of the service are helpful to service users, the long-term effects, appropriateness and cost-effectiveness, and how the service can be integrated into the mental health system.

## Background

In Norway, as in most western countries, various forms of community mental health services have been developed for people experiencing mental distress because of serious mental illness, i.e., community mental health centres (CMHCs), Assertive Community Teams (ACT-teams) and crisis resolution/home treatment teams (CRHT teams). The community mental health services have the promotion of service user involvement as one of their most important underlying principles [1]. Several care models which facilitate user involvement have been developed, ranging from informing service users and their families to user-run services with power-sharing and leadership [2]. Examples of models are patient-centered care, shared decision-making, patient participation models and recovery-oriented care [3]. Over several decades service users and health policy makers have talked a lot about user involvement in mental health care services, yet this rhetoric has largely failed to be translated into practice. A newly published review concluded that services still have not implemented user involvement in the form of empowerment and shared decision making. There is an urgent need to ensure that new service models throughout the mental health service system take account of service users’ views and perspectives [4].

Nowadays more services are provided in the community, but people with serious mental illness may still need for inpatient treatment in times of crisis. Services users emphasise flexible, safe and predictable support during episodes when symptoms are severe and services facilitating coping in phases when they have fewer symptoms [5]. Several CMHCs and mental hospitals in Norway now offer self-referral admissions. This gives a group of service users with serious mental illness who are well-known to services the opportunity to refer themselves for short inpatient stays without contacting their doctor, a duty doctor or emergency department. From the perspective of mental health service providers and policymakers the self-referral admission procedure has the potential to reduce ordinary inpatient stays and crisis admissions to CMHCs and mental hospitals, thus resulting in more appropriate use of inpatient services and lower treatment costs. For service users the opportunity to self-refer is empowering and may thus help to reduce mental distress and improve their quality of life and ability to cope as well as minimising coercion [6].

Empirical research on care models which have implemented a service user involvement strategy is still limited, and robust evidence on the effects of self-referral inpatient admissions is still lacking. This study addressed that gap by exploring the experiences of service users who have had the opportunity to refer themselves for short inpatient stays.

## Methods

### Design and setting

This was a qualitative study based on semi-structured interviews. The study was part of a larger Norwegian randomised controlled trial (RCT) examining effects of self-referral admissions on symptoms, coping, recovery and total use of hospital services. The participants were randomised to two conditions: the intervention group were offered a self-referral admission contract at a CMHC immediately and the control group were offered a similar contract after 1 year (trial registration no. NCT1133587). The contract was a signed, written agreement between the CMHC and the service user giving the service user the power to refer him or herself for a hospital stay of 1–5 days. The contract was an addition to regular treatment. The primary aim during self-referral admissions was to ensure that the stay met the user’s expressed needs e.g., for rest and to restore a stable sleep pattern, managing anxiety or distress or getting out of isolation and having social contact with providers and other service users, rather than on medical and psychological interventions. The providers accepted and respected the user’s decision that he or she needed a short stay in hospital and provided support with practical issues that were of concern to the service user. Further details of the study design are reported elsewhere [6].

### Participants and recruitment

Inclusion criteria for the RCT were experience of periods of mental distress in patients with diagnosis of psychosis with or without substance abuse problems. Patients had to have an ongoing treatment relationship with the CMHC ward taking part in the study and be in receipt of municipal mental health services. Initially, all the participants in the RCT (N = 53) were asked to participate in quarterly, individual semi-structured interviews. The present qualitative interview study included a sample of 28 of these participants who had at least 1 year of experience with a self-referral contract and gave sufficient and relevant information through one or several interviews. The sample represented both the two conditions in the RCT and a total amount of 42 interviews. 14 of the participants were women and the mean age of the sample was 45 years (range: 21–73 years). The participants had one or more diagnoses, including psychosis, mood disorders and substance use. Six of the participants were employed or in education. Further sample details are given in Table 1.

**Table 1:** Characteristics of participants (n = 28) Values are number of participants unless otherwise stated

Characteristic	
Gender	
Male	14
Female	14
Living alone	17
Mean age (range)	45 (21–73)
Diagnosis	
Psychosis (ICD-10; F 20–29)	19
Mood disorders (ICD-10; F 30–39)	8
Substance use (ICD-10; F 10–19)	10
Employment	
Paid work/studies	6
Disability benefits/retirement	21
Sick leave	1
Involuntarily in treatment	10

### Data collection

The interviews followed an initial topic guide. All participants were invited to elaborate on what they did to prevent symptoms and remain stable, their previous and current symptoms and current and previous experiences of mental health services. The flexible interview structure allowed informants to discuss issues of interest to them. At the final interview participants were asked directly about their experiences of self-referral inpatient treatment and were asked to write a brief summary of their experiences. All interviews were conducted in the CMHC or in patients’ homes by experienced psychiatric nurses and lasted between 20 and 60 min. A research associate with user experience participated in some of the interviews. Interviews were carried out between 2010 and 2014. Almost all interviews were audiotaped and transcribed verbatim. A few participants resisted use of tape recorder, data from those consisted of handwritten notes.

### Analysis

The initial analysis was conducted by all the authors—representatives of various mental health professions and a researcher with extensive user experience of mental health services. All transcribed interviews and patients’ written summaries were given a detailed reading and analysed further using systematic text condensation, a general cross-case method for thematic analysis of qualitative data [7]. The analysis consisted of four steps: (1) reading all the material to get an overall impression of preliminary themes; (2) identifying semantic units in the text, i.e., units representing a single aspect of the participant’s relevant experience, and coding them into code groups; (3) clarifying different aspects within the code group, dividing each code group into subgroups from which condensates were developed and illustrative quotations were identified and (4) descriptions of participants’ experiences were extracted. The author group discussed themes, code groups and final categories throughout the analysis process.

### Ethics

The regional committee for medical ethics in East Norway approved the study and it was registered with the Norwegian Data Inspectorate. All participants were given verbal and written information about the study and signed a consent form before taking part in interviews. All participants were given the opportunity to make contact with the staff at the department after the interview. They did not receive any compensation for participating.

## Results

Six participants in the intervention group did not self-refer for hospitalisation during the study period; 22 participants made use of the self-referral admission procedure for one or more stays. The duration of self-referred hospitalisations varied from two to five (the upper limit) days. The number of stays ranged widely, as shown in Table 2. Further details and statistics on use of the self-referral admission procedure will be provided in the forthcoming report from the RCT.

**Table 2:** Frequency of self-referral stays (n = 28)

	No. of self-referral stays				
	0	1	2–4	5–7	8 or more
No. of participants	6	7	5	7	3

Nearly all participants were pleased that they were able to self-refer for a short hospital stay and this was related to something more profound than just the improved access to short hospital stays. The satisfaction was not necessarily connected with experiences from actual use, but its availability through a contract was widely reported to be significant in itself. The results relate to three aspects of self-referral contract experiences: (1) being able to get help or support quickly and easily when it was needed; (2) receiving support adapted to personal needs and (3) being able to make the decision about admission for oneself, without being asked for reasons or being reliant on someone else’s assessment of the severity of one’s difficulties. Detailed results are presented below.

### Easy access to well-known people and frames—a ‘safety valve’

The participants appreciated the low threshold for making contact with the ward and pointed out that being able to get help quickly could avert severe deterioration. Easy access to services and support was also perceived in terms of a reduction in organisational barriers i.e., not having to wait for appointments or contact emergency departments or GPs in order to be admitted to hospital.

Participant (P): It will be a feeling of safety, if something happens I would be certain to get in there without all that hassle… because it is indeed quite a procedure getting admitted. So, yes, it means a lot.

The participants described situations where they had exercised the option to self-refer; these included critical situations such as the death of a close relative or during difficult seasonal holidays. The majority of the participants also cited a perceived increase in well-known symptoms connected to a strong desire to get support early, before further deterioration, as a reason for using the self-admission procedure. Stress, hearing voices, sleep disturbance, an increase in anxiety or more acute changes in their mental condition were commonly mentioned, as well as a general feeling of being in a situation which was too much to handle on one’s own:

P: … it was often related to small episodes, when things are building up….

The option to self-refer was described as a quicker and easier way of seeking support than other admission procedures of which the patients already had experience. But the low threshold for admission was not the only thing which mattered; for patients it was closely connected to the availability and content of treatment or support. Not being forced to wait days or weeks because the bed was in use was highlighted as an important premise. Knowing that one would meet well-known staff on a ward with which one was familiar from earlier stays also contributed to a valuable sense of safety. Not having to tell one’s whole story all over again because one was meeting staff who were familiar with one’s history and having already experienced effective support on the ward also gave patients an important sense of relief and security. Some also mentioned that the CMHC had greater experience and knowledge of their illness and distress than municipal services and said that this made them feel safer. The contract gave patients the option of easy access to a well-known treatment setting and a guarantee that it would be available when needed. The sense of safety afforded by the contract was heavily emphasised; one informant described it as offering a ‘safety valve’:

P: Never used the self-referral opportunity, but have some advantages from it anyway. Having the option to get admitted….. have thought of this option…. gives me a good feeling….. a safety valve.

I: Safety valve, what does that mean?

P: It means that I recall how nice it was here last time…. and it gives a good feeling. A feeling of things moving forward…. a feeling of being taken care of, perhaps…. not having the full responsibility of myself in a way, my own health…. don’t know really.

Getting and signing a contract was something that was taken seriously by the respondents; some said they worked hard on self-governance and self-reflection in order to comply with the agreement. A few mentioned a fear of being dependent on the option to self-refer, and not be able to manage their everyday life on their own. At the same time they made a deliberate effort to limit their use of the service.

### Meeting individual needs—access to a ‘safe haven’

Most of the participants highlighted the importance of having access to a ‘safe haven’ at times of severe mental distress and having treatment that was based on their personal preferences. Personal preferences with regard to the duration and content of the stay were variable. For some 2 days was sufficient, others would like to be able to self-refer for longer than 5 days. Preferences for support and treatment during brief hospital stays under the contract also varied according to the patient and situational factors. For example, some appreciated having an opportunity for dialogue whilst some preferred to be left alone.

P: Yes, you know you have a place to anchor for a little while……. even if things go a bit wrong you still know that you will not be asked up and down and north and south… one is allowed to stay and if you like to talk that’s ok too… at the same time, they will let you alone. I really like that.

The value of being in a safe environment characterised by mutual trust and the feeling of being taken care of contributed to a temporary sense of freedom from responsibility which maximised the restorative effect of the stay.

P: It has been valuable for me when in an acute phase. Getting over the worst period behind safe walls…. Having the chance to go to a safe place and just relax and rest….

Participants found the self-referral procedure as an effective alternative to acute admission or involuntary admission. In their opinion, getting support at an early stage and having access to inpatient treatment which was more tailored to the patient’s personal situation prevented deterioration and obviated the requirement for a longer stay. Hospitalisations under the self-referral contract were commonly characterised by a greater degree of freedom than previous stays; participants compared them favourably with earlier experiences of hospitalisation when someone else had made treatment decisions on their behalf. They appreciated the opportunity that the self-referral option gave them to recover without being subjected to coercion and the fact that it allowed them to continue with their everyday life and carry on being part of society despite requiring hospitalisation.

P: I really believe in this project…. having a place to go to and being looked after before you need to be stuffed with medicines… absolutely, just knowing that you can go to a place where someone will look after you and make sure you get some sleep.

To get well again without… I have been jumped on and stabbed with syringes… it’s crazy, there must be an alternative way to handle this.

Having a restful stay in a safe environment which gave them the opportunity to relax and sleep well restored the participants and they felt these brief hospitalisations filled them with new energy and renewed their motivation to continue coping with mental distress in everyday life.

P: Having a break from a depressing everyday life motivates me to stay off the substance abuse, getting help to take care of myself.

### To be in charge—mutual trust and self-confidence

Participants emphasised the importance of having access to support on the basis of their own judgement of their situation. The fact that they could decide when they needed help and contact the service directly was widely interpreted as a vote of confidence in them by the mental health system. Being capable of judging for themselves when professional support was needed. Many were unfamiliar with the experience of being in charge and having the last word about admission decisions and this gesture of confidence was therefore all the more powerful. The experience of being listened to, believed, taken seriously and given the opportunity to take important decisions for oneself gave patients greater confidence in their coping strategies.

P: … They show me confidence, it’s a system that believes in you…. they trust that I actually need a few days on the ward. When I’m in bad shape, I don’t have to sit for hours in a GP’s surgery….I get taken seriously.

Many emphasised the desire to fend for themselves. The findings indicate that the availability of self-referral encouraged patients to reflect on whether they needed the stay ‘right now’ or could postpone it and ‘wait and see’. They also valued having the option to option to reflect and discuss their decision about a self-referral stay with a well-known member of staff before making their final decision:

P: When I’m feeling bad I get in touch with the mental health service and discuss whether to be admitted or not.

Knowing that they had the option to self-refer at any time made it easier and safer for patients to relax and to make an effort to handle problems at home. This was vital to most of the participants; they expressed a strong wish to be able to manage their condition themselves.

P: … It is actually a bit unusual, but I feel that I can try to weather the storm and get over it at home now. Because I know that if things get too bad I can always make a phone call to check if the self-referral bed is available.

Being in charge of *whether* they need an inpatient stay at a particular point in time and *what* to focus on during the stay was appreciated by the participants and resulted in a relationship of mutual trust between users and the CMHCs. The participants also became more confident in their own judgement and this made it easier both for them to cope with episodes of severe distress at home and to ask for help and support earlier and thus avoid exacerbating their illness.

## Discussion

The findings suggest that self-referral inpatient admissions is more than just a bed. It was experienced as a new, unconventional service which differed substantially from earlier hospitalisations and was characterised by different values and treatment principles. These differences were related to the content, quality and organisation of treatment. Self-referral admission appears to have a positive impact on confidence. Having the option to decide about admission for oneself and having easy access to a service focused on personal needs seemed to enhance service users’ confidence, both in the services they used and in their own ability to cope with everyday life. These findings and their implications for practice will be discussed and related to relevant literature in the sections below.

### Beyond traditional mental health services

Our findings challenge current models of crisis treatment which have a medical focus and are centred on inpatient treatment and rapid stabilisation of acute symptoms [8]. Many of the service users who took part in our study emphasised the benefits of being able to get early help and support through the self-referral contract; something that was missed in earlier studies [9]. Research has reported that there are numerous obstacles to receiving mental health services nowadays; for example lack of accessible support services or patients suffering in silence because they do not recognise their need for help and support [4]. We found that, at least from the point of view of these participants, giving them power to self-refer for a brief hospital stay eliminated earlier perceived barriers to treatment. The self-referral admission contract was received favourably by patients and perceived as a promising initiative related to the strategy to a required reduction in the use of coercion in mental health services. Being able to rely on getting help early in a crisis when they needed made the service users more confidence and made it easier for them to manage their everyday life mental distress and avoid acute admissions and coercive treatment. Similar findings have been reported by another Norwegian pilot study which found a reduction in use of involuntary admissions among service users who signed a self-referral admissions contract [10].

Further, the users in this study did not just contact services because of symptoms directly related to their diagnoses; they also talked about needing a short stay to overcome personal crises. The crises which patients sought to manage through self-referral admissions were not always linked to elevation of symptoms; sometimes they were related to increased stress as a result of larger or smaller events of personal significance. Most research on reasons for mental health admissions has tried to relate psychiatric hospitalisation to clinical variables and rather little attention has been paid to users’ opinions of when and why they need help and support. It has also been pointed out that there is a lack of research methods suitable for investigating the complex situational variables associated with mental health inpatient treatment [11].

### Individual care and support

Participants in our study reported that they had been offered personalised help and support when they had used the self-referral inpatient admission procedure. They appreciated and found it helpful to have an early opportunity to discuss the management of their immediate problems and coping strategies for everyday life with health care professionals with whom they had an established relationship. Our findings are consistent with the large body of evidence that there is a need for mental health services to adapt to the needs of users rather than vice versa, and that mental health service users should be involved in planning their own care [4]. Person-centred services are one way of making service users and their families partners in the planning, development and evaluation of mental health services [12, 13]. Research on person-centered practices has reported results accordance between mental health care providers and service users on treatment plans, improved health outcomes and increased user satisfaction [14]. Recovery oriented services are another way of involving service users in decisions about the management of their illness, this model involves supporting recovery by promoting hope and optimism in service users, encouraging them to develop a positive identity, find meaning in life and empowering them [15, 16].

Participants in this study expressed their gratitude for the opportunity to participate in the project and the chance it had given them to be in the driver’s seat when it came to taking important decisions about their treatment and care. For them, being able to decide *when* they needed an inpatient stay and *what* to focus on during that stay was very different from earlier experiences of mental health services. Being ‘in charge’ in this way had other positive effects, for example on patients’ self-confidence, which corroborate other research suggesting that allowing people with mental health problems to become actively involved in their own care has therapeutic benefits [17–19]. There is evidence of associations between user involvement and improved self-esteem [17] and empowerment [20–23]. Another study involving participants recruited from the larger RCT study also found that users with a self-referral contract had more confidence in own coping strategies than users with no contract [6], but other studies reported that the effects of user participation in treatment planning are inconsistent and the implication unclear [24]. One explanation for the inconsistencies in the evidence is that there is no standard definition of user involvement and that validated instruments for investigating its effects are lacking [17].

In summary, the users in this study appreciated having access to inpatient crisis services with a strong focus on user involvement. Similar findings are reported from CRHT teams, which also emphasize user involvement [25, 26]. It is clear that the positive experiences reported in our study were critically dependent on the way in which self-referral admissions were handled; it was important to our participants that they were admitted to a unit where they were known and that they could rely on a bed being available when they needed it.

### Strengths and limitations

This study was based on data from a substantial sample of service users who reported their experiences of self-referral hospitalisation; the sample was substantially larger than is usual for a study of this type. Robust qualitative methodology was used. It is possible, however that we lost important data because of the relatively short study period. Some participants had not made use of the self-referral admission procedure before the end of the data collection period, but reported they thought they probably would take advantage of the option in the longer term. Most of the interviews were conducted by researchers who previously worked as psychiatric nurses at the CMHS where the study was based. Their relationship with the CMHS might have resulted in more favourable evaluations of the self-referral service than if the interviews had been conducted by independent researchers.

## Conclusion

Self-referral inpatient admission is a concrete example of user involvement in mental health services. Users perceived it as a service that went beyond the traditional mental health service approach and reported that it embodied values and treatment principles more like those of community mental health care than institutional care. It is nevertheless important to emphasise that the self-referral admission service described here is an offer in development and that we await the findings of the larger RCT study. More knowledge is needed about the content of the service and which aspects of it are important to what kinds of service user, as well as evidence on long-term outcomes, cost-effectiveness and how the service can be integrated into the mental health system.

## References

[1] Thornicroft G, Alem A, Dos Santos RA, Barley E, Drake RE, Gregorio G (2010). WPA guidance on steps, obstacles and mistakes to avoid in the implementation of community mental health care. World Psychiatry.

[2] Wallcraft J, Amering M, Freidin J, Davar B, Froggatt D, Jafri H (2011). Partnerships for better mental health worldwide: WPA recommendations on best practices in working with service users and family carers. World Psychiatry.

[3] Storm M, Edwards A (2013). Models of user involvement in the mental health context: intentions and implementation challenges. Psychiatr Q.

[4] Newman D, O’Reilly P, Lee SH, Kennedy C (2015). Mental health service users’ experiences of mental health care: an integrative literature review. J Psychiatr Ment Health Nurs.

[5] Rise MB, Westerlund H, Bjørgen D, Steinsbekk A. Safely cared for or empowered in mental health care? Yes, please. Int J Social Psychiatry. 2013:0020764012471278. 10.1177/0020764012471278 23321388

[6] Rise M, Evensen G, Moljord I, Ro M, Bjorgen D, Eriksen L (2014). How do patients with severe mental diagnosis cope in everyday life—a qualitative study comparing patients’ experiences of self-referral inpatient treatment with treatment as usual?. BMC Health Serv Res.

[7] Malterud K (2012). Systematic text condensation: a strategy for qualitative analysis. Scand J Public Health.

[8] Sharfstein SS (2009). Goals of inpatient treatment for psychiatric disorders. Annu Rev Med.

[9] Gudde CB, Olsø TM, Antonsen DØ, Rø M, Eriksen L, Vatne S (2013). Experiences and preferences of users with major mental disorders regarding helpful care in situations of mental crisis. Scand J Public Health.

[10] HeskestadSaT M (2008). Patient-guided crisis admissions for severe psychotic conditions. Tidsskr Nor Laegeforen.

[11] Chai YK, Wheeler Z, Peter H, Gale C, Glue P (2013). Factors associated with hospitalization of adult psychiatric patients: cluster analysis. Australas Psychiatry.

[12] McCormack B, McCance T (2011). Person-centred nursing: theory and practice.

[13] McCance T, McCormack B, Dewing J (2011). An exploration of person-centredness in practice. Online J Issues Nurs.

[14] Ekman I, Swedberg K, Taft C, Lindseth A, Norberg A, Brink E (2011). Person-centered care—Ready for prime time. Eur J Cardiovasc Nurs.

[15] Leamy M, Bird V, Le Boutillier C, Williams J, Slade M (2011). Conceptual framework for personal recovery in mental health: systematic review and narrative synthesis. Br J Psychiatry.

[16] Bonney S, Stickley T (2008). Recovery and mental health: a review of the British literature. J Psychiatr Ment Health Nurs.

[17] Stringer B, Van Meijel B, De Vree W, Der Van, Bijl J (2008). User involvement in mental health care: the role of nurses. A literature review. J Psychiatr Ment Health Nurs.

[18] Ward PR, Thompson J, Barber R, Armitage CJ, Boote JD, Cooper CL (2010). Critical perspectives on ‘consumer involvement’in health research epistemological dissonance and the know-do gap. J Sociol.

[19] McCann T, Baird J, Clark E, Lu S (2008). Mental health professionals’ attitudes towards consumer participation in inpatient units. J Psychiatr Ment Health Nurs.

[20] Tambuyzer E, Van Audenhove C (2013). Is perceived patient involvement in mental health care associated with satisfaction and empowerment?. Health Expect.

[21] Simpson E, House A (2003). User and carer involvement in mental health services: from rhetoric to science. Br J Psychiatry.

[22] van de Bovenkamp H, Trappenburg M (2009). Reconsidering patient participation in guideline development. Health Care Anal.

[23] Felton A, Stickley T (2004). Pedagogy, power and service user involvement. J Psychiatr Ment Health Nurs.

[24] Tambuyzer E, Pieters G, Van Audenhove C (2014). Patient involvement in mental health care: one size does not fit all. Health Expect.

[25] Karlsson B, Borg M, Kim HS (2008). From good intentions to real life: introducing crisis resolution teams in Norway. Nurs Inq.

[26] Barker V, Taylor M, Kader I, Stewart K, Le Fevre P (2011). Impact of crisis resolution and home treatment services on user experience and admission to psychiatric hospital. Psychiatr.

